# Baseline Chloride Levels are Associated with the Incidence of Contrast-Associated Acute Kidney Injury

**DOI:** 10.1038/s41598-017-17763-7

**Published:** 2017-12-12

**Authors:** Hyung Jung Oh, Sungwon Kim, Jung Tak Park, Sang-Joon Kim, Seung Hyeok Han, Tae-Hyun Yoo, Dong-Ryeol Ryu, Shin-Wook Kang, Yong Eun Chung

**Affiliations:** 1 Ewha Institute of Convergence Medicine, Ewha Womans University Mokdong Hospital, Seoul, Republic of Korea; 2 Research Institute for Human Health Information, Ewha Womans University Mokdong Hospital, Seoul, Republic of Korea; 3 Department of Radiology, Yonsei University College of Medicine, 50-1 Yonsei-ro, Seodaemun-gu, Seoul, 03722 Korea; 4 Department of Internal Medicine, College of Medicine, Yonsei University, Seoul, Republic of Korea; 5 Ewha School of Business, Ewha Womans University, Seoul, Republic of Korea; 6 Department of Internal Medicine, School of Medicine, Ewha Womans University, Seoul, Republic of Korea; 7 Tissue Injury Defense Research Center, Ewha Womans University, Seoul, Republic of Korea; 8 BK21 PLUS Project for Medical Science, Yonsei University College of Medicine, Seoul, Republic of Korea

## Abstract

Although hypo- and hyperchloremia have been associated with worsening renal outcomes, there has been no study that correlates hypo- and hyperchloremia and the incidence of contrast-associated acute kidney injury (CA-AKI). A total of 13,088 patients with less than 2.0 mg/dL of serum creatinine (Cr) who underwent contrast-enhanced abdominal CT (CECT) were included. Patients were divided into 3 groups based on Cl (the hypo-, normo- and hyperchloremia groups). Patients were also classified by baseline Cr (<1.2; the ‘Normal Cr group’ and 1.2–2.0 mg/dL; the ‘Slightly increased Cr group’). Multivariate logistic regression analysis was used to reveal the association between Cl and CA-AKI. Among patients, 2,525 (19.3%) and 241 (1.8%) patients were classified in the hypo- and hyperchloremia group. The incidence of CA-AKI was significantly lower in the normochloremia group (4.0%) compared to the hypo- (5.4%) and hyperchloremia groups (9.5%). On multivariate logistic regression, hypochloremia was significantly associated with the incidence of CA-AKI compared with normochloremia (1.382, P = 0.002). Moreover, hypochloremia was still significantly associated with the incidence of CA-AKI in ‘Normal Cr group’ compared with normochloremia (1.314, P = 0.015), while hyperchloremia did not show significant association with CA-AKI incidence. In conclusion, hypochloremia might be associated with the incidence of CA-AKI even in patients who have normal-range Cr levels.

## Introduction

Acute deterioration of renal function due to iodinated contrast agents, defined as contrast-associated acute kidney injury (CA-AKI), is generally mild and transient, but sometimes can result in lasting renal dysfunction and need for renal replacement therapy^1–3^. CA-AKI is the leading cause of new onset renal failure in hospitalized patients, with the highest risk being observed in patients with pre-existing impaired renal function^1–3^, and it is significantly associated with increases in in-hospital and long-term morbidity and mortality, acceleration of chronic renal disease, and increased costs for medical care^4^.

Although the pathophysiology of CA-AKI is poorly understood, it may include acute vasoconstriction resulting in renal hypoperfusion, hypoxia-induced oxidative stress, and free radicals generated within the acid environment of the renal medulla^5^. However, since there is no specific therapy for CA-AKI and because the disease is mostly iatrogenic, prevention is considered to be of paramount importance, and a variety of approaches have been suggested to prevent CA-AKI by targeting these pathomechanisms^6–11^; 24-hour volume supplementation with sodium chloride 0.9% is one approach still accepted and it is considered a cornerstone in the prevention of CA-AKI in clinical practice^12,13^. However, sodium chloride saline is chloride-rich^14–16^, and supplementing patients with the solution can lead to hyperchloremia after fluid replacement^16,17^. Also, the consequent hyperchloremic metabolic acidosis derived from chloride-rich solutions has been associated with poorly progressive outcomes in critically ill patients^16,18–20^.

Hypochloremia has also been independently associated with increased risk of mortality in critically ill patients, and this has been explained with the occurrence of metabolic alkalosis^21^. However, there are only a few observational studies evaluating the association of hypochloremia with hospital morbidity^22,23^. Moreover, no studies have been done on the correlation between hypochloremia and the incidence of CA-AKI. Thus, the aim of this study was primarily to investigate the association between the incidence of CA-AKI and baseline serum chloride concentrations by stratifying patients into three groups (hypochloremia, normochloremia, and hyperchloremia). Secondarily, we evaluated how changes in chloride levels from the baseline level affected the incidence of CA-AKI for each group.

## Results

### Baseline characteristics

The mean age was 59.0 ± 15.3 years, and 7,635 (58.3%) patients were male. In addition, the mean body mass index (BMI) was 22.7 ± 3.5 kg/m^2^, and the detailed demographics are summarized in Table 1.

**Table 1:** Baseline characteristics among the patients performing contrast CT, but with less than 2.0 mg/dL of serum creatinine at baseline.

**Variables**	**Total (n = 13,088, 100%)**	**Hypochloremia (n = 2,525, 19.3%)**	**Normochloremia (n = 10,322, 78.9%)**	**Hyperchloremia (n = 241, 1.8%)**	**p-value**
Age, years	59.0 ± 15.3	60.0 ± 14.8	58.7 ± 15.4	64.2 ± 15.9	<0.001
Male, n (%)	7,635 (58.3%)	1,504 (59.6%)	6,011 (58.2%)	120 (49.8%)	0.012
BMI, kg/m^2^	22.7 ± 3.5	22.2 ± 3.6	22.9 ± 3.4	22.8 ± 4.1	<0.001
Inpatients, n (%)	11539 (88.2%)	2391 (94.7%)	8913 (86.3%)	235 (97.5%)	<0.001
Comorbid disease, n(%)					
DM	3,800 (29.0%)	873 (34.6%)	2,841 (27.5%)	86 (35.7%)	<0.001
Hypertension	5,881 (44.9%)	1,182 (46.8%)	4,559 (44.2%)	140 (58.1%)	<0.001
Dyslipidemia	2,361 (18.0%)	452 (17.9%)	1,855 (18.0%)	54 (22.4%)	0.205
CAD	1,368 (10.5%)	266 (10.5%)	1,055 (10.2%)	47 (19.5%)	<0.001
Heart failure	506 (3.9%)	104 (4.1%)	386 (3.7%)	16 (6.6%)	0.053
Contrast volume, mL	120.5 ± 20.0	117.3 ± 20.9	121.4 ± 19.6	118.1 ± 22.3	<0.001
Laboratory data					
BUN, mg/dL	16.1 ± 8.6	17.1 ± 9.6	15.6 ± 8.1	23.4 ± 13.7	<0.001
Creatinine, mg/dL	0.76 ± 0.26	0.74 ± 0.27	0.76 ± 0.26	0.84 ± 0.32	<0.001
Sodium, mEq/L	137.5 ± 4.5	132.0 ± 4.5	138.7 ± 3.1	145.1 ± 5.1	<0.001
Chloride, mEq/L	101.1 ± 4.9	93.9 ± 3.5	102.5 ± 2.9	113.6 ± 3.8	<0.001
tCO2, mEq/L	23.8 ± 3.6	24.4 ± 4.1	23.7 ± 3.3	20.5 ± 4.3	<0.001
CA-AKI rate, n (%)	568 (4.3%)	137 (5.4%)	408 (4.0%)	23 (9.5%)	<0.001

Data are expressed as mean (with standard deviation) or n (%); Abbreviations; DM, diabetes mellitus; CAD, coronary arterial disease; BUN, blood urea nitrogen; tCO_2_, total CO_2_; CA-AKI, contrast-associated acute kidney injury.

Hypochloremia; chloride level less than 98 mEq/L at baseline; Normochloremia; chloride level between 98 to 110 mEq/L at baseline; Hyperchloremia; chloride level over 110 mEq/L at baseline.

**Figure 1:**
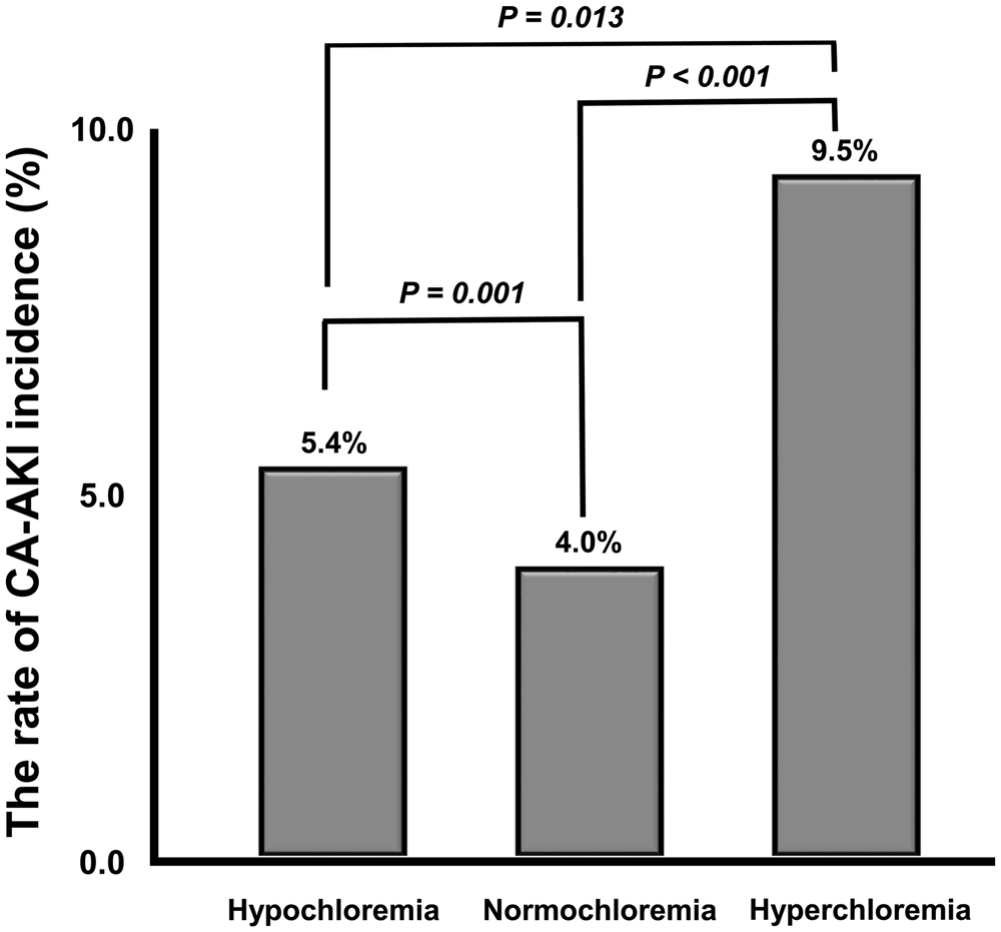
The rate of CA-AKI incidence. CA-AKI incidence was the highest in the hyperchloremia group (9.5%), and the second highest in the hypochloremia group (5.4%), with statistically significance compared to the normochloremia group (4.0%). Moreover, there was also significant difference in the incident rate of CA-AKI between the hypochloremia and hyperchloremia groups Abbreviations; CA-AKI, contrast-associated acute kidney injury.

When these patients were divided into three groups (hypochloremia, normochloremia, and hyperchloremia), 2,525 (19.3%) patients were in the hypochloremia group, and 241 (1.8%) patients were in the hyperchloremia group. Most of the patients were inpatients [11539 (88.1%)], and the hyperchloremia group had the highest portion of inpatients (94.7%) among the three groups with statistical significance. Patients in the hyperchloremia group were significantly older, and had more diabetes mellitus (DM), hypertension, and coronary artery disease (CAD) compared to the other groups, and there were significantly more males in the hypochloremia group compared to the other groups. In the normochloremia group, the BMI was significantly higher than the other groups. The administered volume of contrast media was also significantly different between the groups. However, the volume per weight was the same for all groups because the contrast media volume was adjusted to each patient’s body weight (2 mL/kg). Moreover, of the laboratory data, serum blood urea nitrogen (BUN), creatinine (Cr), sodium, and chloride concentrations were significantly increased in the hyperchloremia group, whereas the serum total CO_2_ level was significantly elevated in the hypochloremia group compared to the other groups (Table 1). In addition, CA-AKI incidence was the highest in the hyperchloremia group (9.5%), and the second highest in the hypochloremia group (5.4%), with statistically significance compared to the normochloremia group (4.0%). Moreover, there was also significant difference in the incident rate of CA-AKI between hypochloremia and hyperchloremia groups (Table 1 and Fig. 1).

### Hypochloremia is associated with incidence of CA-AKI

**Figure 2:**
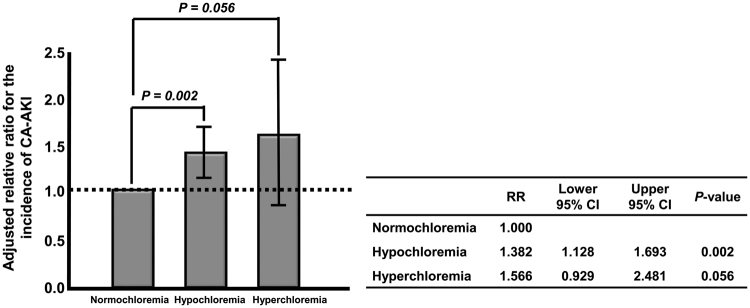
Adjusted relative risk of CA-AKI. Hypochloremia was significantly associated with CA-AKI incidence compared to normochloremia even after adjusting for several covariables, whereas hyperchloremia was marginally related to CA-AKI incidence compared to normochloremia. Abbreviations; CA-AKI, contrast-associated acute kidney injury.

Univariate analysis showed that both hypochloremia (RR; 1.394, 95% CI; 1.143–1.700, P = 0.001) and hyperchloremia (2.561, 95% CI; 1.650–3.984, P < 0.001) were significantly related to increased CA-AKI incidence compared to normochloremia. In addition, hypochloremia was significantly associated with CA-AKI incidence compared to normochloremia even after adjusting for several covariables [relative risk (RR), 1.382; 95% confidence interval (CI), 1.128–1.693; P = 0.002], whereas hyperchloremia was marginally related to the CA-AKI incidence compared to normochloremia (RR, 1.566; 95% CI, 0.929–2.481; P = 0.056) (Table 2 and Fig. 2).

**Table 2:** Univariate and multivariate logistic regression analysis for the incidence of CA-AKI on the baseline chloride level (n = 13,088).

**Variables**	**Univariate**		**Multivariate**	
	**RR (95% CI)**	**P-value**	**RR (95% CI)**	**P-value**
Normochloremia	Reference		Reference	
Hypochloremia	1.394 (1.143–1.700)	0.001	1.382 (1.128–1.693)	0.002
Hyperchloremia	2.564 (1.650–3.984)	<0.001	1.566 (0.989–2.481)	0.056

Abbreviations; RR, relative risk; CI, confidence interval; CA-AKI, contrast-associated acute kidney injury.

Adjusted for age, sex, admission status, comorbidity diseases including DM, hypertension, coronary artery disease, and heart failure, serum creatinine level, serum BUN level, serum tCO2.

Hypochloremia; chloride level less than 98 mEq/L at baseline.

Normochloremia; chloride level between 98 to 110 mEq/L at baseline.

Hyperchloremia; chloride level over 110 mEq/L at baseline.

### The incidence of CA-AKI according to creatinine levels

The same analyses were conducted in patients who had serum Cr concentrations of less than 1.2 mg/dL (the ‘Normal Cr group’, n = 12,280) or 1.2 to 2.0 mg/dL (the ‘Slightly increased Cr group’, n = 808) serum Cr concentrations at baseline, respectively, and the baseline characteristics are described in Supplementary Tables 1 and 2.

Multivariate logistic analyses presented that hypochloremia was still significantly associated with CA-AKI incidence in the ‘Normal Cr group’ compared with normochloremia (RR, 1.314; 95% CI, 1.550–1.636; P = 0.015), while hyperchloremia did not show significant association with the CA-AKI incidence in ‘Normal Cr group’. In ‘Slightly increased Cr group’, neither hypochloremia nor hyperchloremia was significantly related to CA-AKI incidence (Table 3).

**Table 3:** Subgroup analysis for the incidence of CA-AKI on the baseline chloride level depending on serum creatinine level [‘Normal Cr group’ (n = 12,280) and ‘Slightly increased Cr group (n = 808)].

Variables	Univariate		Multivariate	
	RR (95% CI)	P-value	RR (95% CI)	P-value
Normal Cr group (n = 12,280)				
Normochloremia	Reference		Reference	
Hypochloremia	1.347 (1.086–1.671)	0.007	1.314 (1.550–1.636)	0.015
Hyperchloremia	2.356 (1.419–3.913)	0.001	1.360 (0.799–2.315)	0.257
Slightly increased Cr group (n = 808)				
Normochloremia	Reference		Reference	
Hypochloremia	1.781 (1.048–3.027)	0.033	1.641 (0.940–2.866)	0.082
Hyperchloremia	2.303 (0.912–5.819)	0.078	2.322 (0.872–6.186)	0.092

Abbreviations; CA-AKI, contrast-associated acute kidney injury; RR, relative risk; CI, confidence interval; Cr, creatinine.

Adjusted for age, sex, admission status, comorbidity diseases including DM, hypertension, coronary artery disease, and heart failure, serum creatinine level, serum BUN level, serum tCO2.

Normal Cr group; patients who had a serum Cr level of less than 1.2 mg/dL.

Slightly increased Cr group; patients who had 1.2 ≤ serum Cr levels < 2.0 mg/dL.

Hypochloremia; chloride level less than 98 mEq/L at baseline.

Normochloremia; chloride level between 98 to 110 mEq/L at baseline.

Hyperchloremia; chloride level over 110 mEq/L at baseline.

### Effect of increases in chloride concentration on the incident rate of CA-AKI

We examined the post chloride concentration when the post Cr was measured and calculated delta chloride (ΔCl). Positive ΔCl, defined by an increase in chloride concentration from the baseline concentration, was significantly associated with a reduction in CA-AKI incidence in all groups of hypochloremia (in both the ‘Normal Cr group’ and the ‘Slightly increased Cr group’) compared to negative ΔCl (in the ‘Normal Cr group’, RR, 0.318; 95% CI, 0.193–0.527; P < 0.001, in the ‘Slightly increased Cr group’, RR, 0.053; 95% CI, 0.009–0.326; P = 0.002), while in hyperchloremia group, positive ΔCl was significantly related to an increase in CA-AKI incidence in the ‘Normal Cr group’ compared to negative ΔCl (RR, 3.092; 95% CI, 1.080–10.655; P = 0.036) (Table 4).

**Table 4:** Multivariate logistic regression analyses for the incidence of CA-AKI with delta chloride concentration within 72 hours in hypo- and hyperchloremia groups.

Variables	Hypochloremia		Hyperchloremia	
	RR (95% CI)	P-value	RR (95% CI)	P-value
**Normal Cr group (positive** Δ**Cl vs. negative** Δ**Cl)**	0.318 (0.193–0.527)	< 0.001	3.092 (1.080–10.655)	0.036
**Slightly increased Cr group (positive** Δ**Cl vs. negative** Δ**Cl)**	0.053 (0.009–0.326)	0.002	—	—

Abbreviations; CA-AKI, contrast-associated acute kidney injury; RR, relative risk; CI, confidence interval; Cr, creatinine.

ΔCl was defined by [post peak chloride concentration – chloride concentration at baseline].

Positive ΔCl was defined as ΔCl ≥ 0, while negative ΔCl was defined as ΔCl < 0.

Adjusted for age, sex, BMI, admission status, comorbidity diseases (such as DM, hypertension, coronary artery disease, and heart failure), serum creatinine, serum BUN, tCO2, and contrast media volume.

Normal Cr group; patients who had a serum Cr level of less than 1.2 mg/dL.

Slightly increased Cr group; patients who had 1.2 ≤ serum Cr levels < 2.0 mg/dL.

Hypochloremia; chloride level less than 98 mEq/L at baseline.

Normochloremia; chloride level between 98 to 110 mEq/L at baseline.

Hyperchloremia; chloride level over 110 mEq/L at baseline.

## Discussion

Several previous studies described a positive correlation between chloride levels and severity of AKI^19,20,24^. Especially, Yunos *et al*.^24^ demonstrated that chloride-restrictive solution results in a lower incidence of AKI compared with chloride-liberal fluid strategies in critically ill patients. However, recent studies could not prove that sodium bicarbonate was superior to isotonic sodium chloride for preventing incidence of CA-AKI^25,26^. Therefore, we tried to identify the association between CA-AKI incidence and baseline serum chloride concentrations and to evaluate what effect an increase in chloride level would have on the incidence of CA-AKI. In the current study, the rate of CA-AKI incidence was significantly increased in the hypochloremia group by 38.2% compared to the normochloremia group, and was marginally increased in the hyperchloremia group compared to the normochloremia group. In contrast, hypochloremia was significantly associated with an increase in CA-AKI incidence only in the ‘Normal Cr group’, while there were no significant associations between incidence of CA-AKI incidence and hyperchloremia in the ‘Normal Cr group’. Plus, neither hypochloremia nor hyperchloremia was significantly related to CA-AKI incidence in the ‘Slightly increased Cr group’. However, positive ΔCl was significantly associated with reduced CA-AKI incidence in the hypochloremia group compared with negative ΔCl, whereas it was significantly related to increased CA-AKI incidence in the hyperchloremia group of the ‘Normal Cr group’.

Hypochloremia is related to mortality in critically ill patients, and this is thought to be due to metabolic alkalosis^27,28^, but the main factor responsible for mortality due to hypochloremia and metabolic alkalosis is unclear^23,29^. Tani *et al*.^23^ showed that hypochloremic patients with metabolic acidosis had a higher mortality rate compared with those with metabolic alkalosis, suggesting that hypochloremia is associated with mortality independently of metabolic alkalosis. Moreover, hypochloremia could be a sign of illness severity as a result of dysregulated homeostasis^22^, and it can be related to increased CA-AKI incidence compared with normochloremia. In this study, patients with hypochloremia were also significantly older, and had more DM and hypertensive comorbidity diseases than patients with normochloremia. However, hypochloremia was still independently associated with increased CA-AKI incidence even after adjusting for the above factors, suggesting that hypochloremia was associated with an increase in CA-AKI incidence, independent of the disease comorbidity. Unfortunately, we could not measure serial total CO_2_ levels in these patients to evaluate changes in acid-base. This is thought to be one of several limitations that can explain why positive ΔCl helped to reduce the incidence of CA-AKI in the hypochloremia group, but aggravated the incident rate of CA-AKI in hyperchloremia group compared with negative ΔCl. However, based on our results, active correction of serum chloride levels before contrast-enhanced CT examination might help prevent CA-AKI incidence in hypochloremic milieu.

In comparison, hyperchloremia was not related to the incidence of CA-AKI in this study. Unlike previous studies, the hyperchloremia group was comprised of only 248 (1.8%) patients, hampering identification of significant associations between hyperchloremia and the increased CA-AKI incident rate. Thus, further studies involving larger populations are warranted to determine the relationship between hyperchloremia and CA-AKI. In contrast, since positive ΔCl was significantly related to an increase of CA-AKI incidence compared with negative ΔCl in the hyperchloremia group of the ‘Normal Cr group’, caution should be taken not to elevate chloride levels when patients are in a hyperchloremic condition, even though they might need contrast CT examinations.

Although alkalization with bicarbonate perfusion may attenuate the production of hydroxyl radicals and thus reduce the formation of reactive oxygen species^30^, bicarbonate solution cannot be determined as superior to saline solution for preventing CA-AKI incidence^25,26^. However, this study can suggest that additional interventional studies to investigate the superiority of each solution (bicarbonate solution vs. saline solution) may be useful after patients are stratified according to baseline chloride levels.

As seen in Supplementary Table 3, the ‘No CA-AKI group’ used a significantly higher total contrast volume compared to the ‘CA-AKI group’ in the ‘Slightly increased Cr group’, while there were no significant differences in the total contrast volume used between the two groups in all the enrolled patients and the ‘Normal Cr group’. However, since the amount of contrast agent injected is adjusted to each patient’s body weight (2 ml/kg), the administered contrast volume per body weight (kg) was the same throughout this study. The difference in total contrast volume between the three groups of patients with Cr of 1.2 to 2.0 mg/dL might originate from different body weights.

There are several limitations in this study. First, this was retrospective cohort study and thus was subject to selection bias. Second, patients were arbitrarily classified into three groups according to our hospital reference values, and consequently, patients were distributed unevenly among the three groups. However, there are no established cut-off values for hypo-, normo-, or hyperchloremia^19,22,23^; most studies use cut-off values of 98 mEq/L for hypochloremia and 110 mEq/L for hyperchloremia^19,22^. Third, only patients with a serum Cr concentration of less than 2.0 mg/dL were selected arbitrarily. Thus, our findings cannot be extrapolated beyond 2.0 mg/dL of serum Cr. Fourth, the current status of patients and the reasons for CT examination were not investigated. Fifth, we did not investigate how acid‒base changed according to alterations of the chloride level, and it was hard to explain the pure effect of chloride level changes on preventing CA-AKI. Despite these limitations, for the first time, this study showed that hypochloremia may be independently associated with increased CA-AKI incidence especially in patients with normal kidney function, and that an increase in chloride level from the baseline level may be independently related to decrease of CA-AKI incidental rate in hypochloremic patients compared with a decrease in chloride level from the baseline, while in hyperchloremia, elevation of chloride rather increased the CA-AKI incidence.

In conclusion, hypochloremia might be significantly associated with increased CA-AKI especially in patients with normal kidney function and an increment of chloride might reduce the incidence of CA-AKI in hypochloremic conditions. Hence, patients with hypochloremia and normal renal function at baseline should be monitored closely when they need to undergo a contrast-CT examination. In the future, a randomized-controlled interventional study involving a larger population is needed to evaluate the association between increased chloride levels and the CA-AKI incident rate of hypochloremic patients.

## Materials and Methods

### Study population

This retrospective and Health Insurance Portability and Accountability Act-compliant (HIPAA) study was approved by the institutional review board of Yonsei University College of Medicine and informed consent was waived. This study was performed in accordance with HIPAA. From January 2014 to December 2015, patients who met the following inclusion criteria were included in this study: 1) patients who underwent contrast-enhanced abdominal CT, 2) patients who had laboratory results including serum BUN, Cr and Cl levels within 1 month before CT examination and within 72 hours after CT examination. Through a search of the electronic medical database, 14,069 patients who met the inclusion criteria were found. Among them, patients who did not have laboratory results for the total CO_2_ level or the serum sodium level within 1 month before CT examination (n = 315) were excluded.

**Figure 3:**
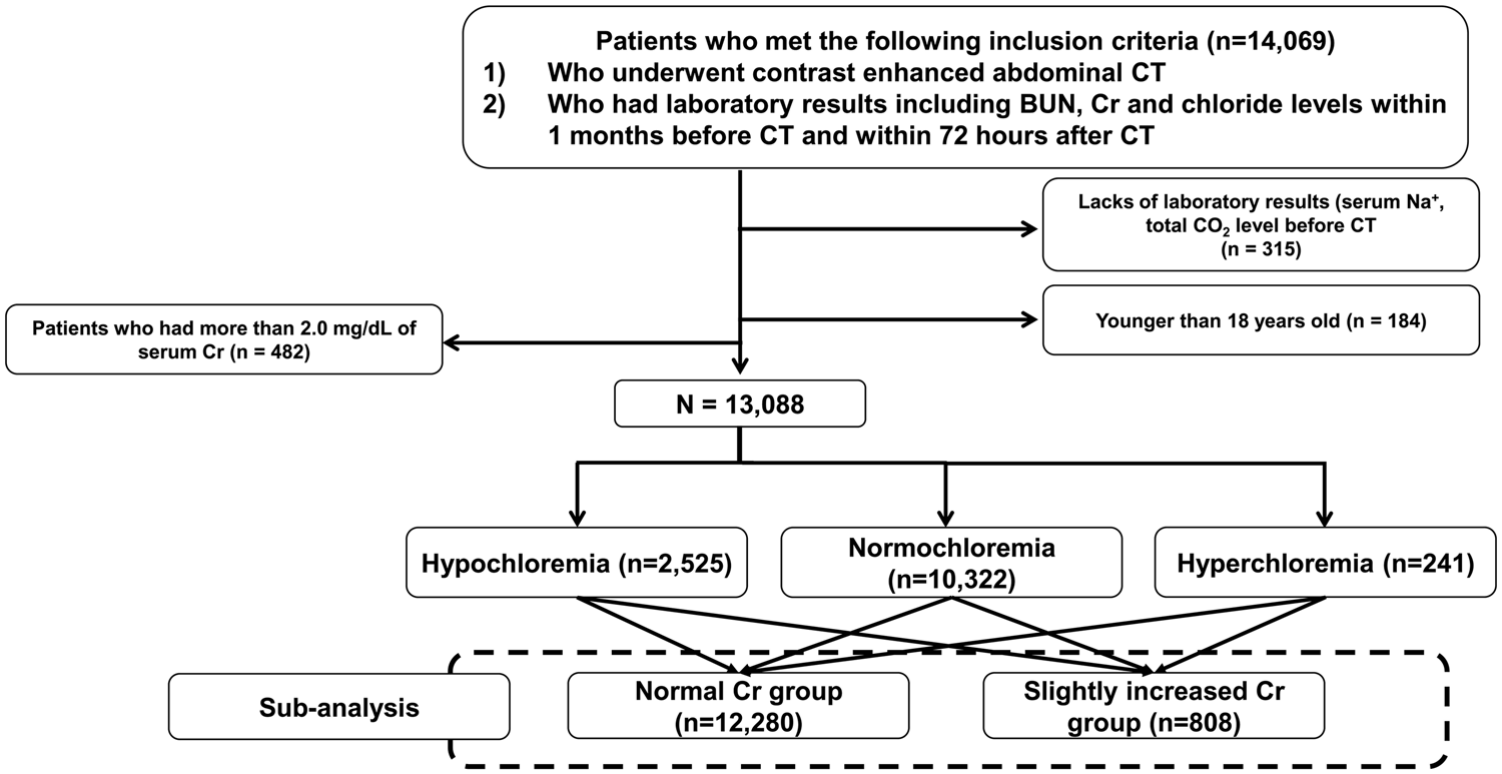
Flow chart of patients’ inclusion and subgrouping.

Additionally, 184 patients who were younger than 18 years old, and 482 patients who had more than 2.0 mg/dL of serum Cr before CT examination were also excluded. Finally, 13,088 patients (mean age, 59.0 ± 15.3; male ratio, 58.3%) were included in this study (Fig. 3). Patients were divided into three groups according to the reference values of our institution: the hypochloremia group (serum Cl^−^ < 98 mEq/L, n = 2,525), normochloremia group (98 ≤ serum Cl^−^ ≤ 110 mEq/L, n = 10,322), and hyperchloremia group (serum Cl^−^ > 110 mEq/L, n = 241).

### Data collection

Data on baseline characteristics, including demographic information and preexisting chronic comorbidities were collected at the point of CT examination. Moreover, baseline laboratory data acquired within 1 month before CT examination were collected, and the peak values of post BUN and Cr concentrations were recorded within 72 hours after performing CT. In this study, we considered the last values obtained before the patient underwent CT as the baseline laboratory data, but we excluded patients who did not have laboratory results within 1 month before the CT procedure. We theorized that the last value obtained before CT examination would reflect each patient’s condition better than other laboratory values. Most of the inpatients in the current study had laboratory results obtained within 3 days before CT examination, and we assumed that these results would effectively show the status of each patient before CT procedure. Meanwhile, according to our hospital policy, laboratory results (especially BUN and Cr) obtained within 1 month before CT examination are considered as recent data for outpatients, while patients who do not have BUN/Cr results obtained within 1 month before CT procedure undergo laboratory tests. Hence, we set the ‘1 month before CT examination as the cut-off timeline for recent laboratory data. In addition, serum chloride concentrations were measured by indirect potentiometry (ADVIA 1800 Chemistry System; Siemens Healthcare Diagnostic Inc., Oakville, ON, Canada). We also measured chloride concentrations at the same time as serum post BUN and Cr concentrations to evaluate how changes in chloride levels affected the development of CA-AKI.

### Definition

1. CA-AKI was defined based on the KDIGO criteria with any of the following conditions; Increase in SCr by ≥0.3 mg/dL within 48 hours or increase in SCr to ≥1.5 times baseline, which is known or presumed to have occurred within the prior 7 days or urine volume < 0.5 mL/kg/h for 6 hours^31^.
2. Post BUN was defined as the peak serum BUN concentration found within 72 hours after CT examination
3. Post Cr was defined as the peak serum Cr concentration found within 72 hours after CT examination
4. Delta creatinine level (ΔCr) (%) was defined by [Cr_within 72h_ − Cr_at baseline_]/[Cr_at baseline_] × 100(%).
  Cr_within 72h_ was defined by the peak value of serum Cr found within 72 hours after contrast administration.
  Cr_at baseline_ was defined as the serum Cr level found before contrast administration.
5. Delta chloride level (ΔCl) (mEq/L) was defined by [Cl^−^_within 72h_ − Cl^−^_at baseline_].
  Cl^−^_within 72h_ was defined by the peak value of chloride found within 72 hours after contrast administration.
  Cl^−^_at baseline_ was defined as the chloride level found before contrast administration.
  Positive ΔCl was defined as ΔCl ≥ 0, while negative ΔCl was defined as ΔCl < 0.

### Sub-analysis

All patients were separated into two groups with the normal reference range of serum Cr (1.2 mg/dL) used in our hospital; the ‘Normal Cr group’ had a serum Cr level of less than 1.2 mg/dL (n = 12,280) and the ‘Slightly increased Cr group’ had 1.2 ≤ serum Cr levels < 2.0 mg/dL (n = 808). We analyzed the associations between hypo- or hyperchloremia and the increase in CA-AKI occurrence for the ‘Normal Cr group’ and the ‘Slightly increased Cr group’, respectively. Moreover, all patients were also divided into two groups according to changes in chloride concentrations before and after CT examination: positive ΔCl and negative ΔCl. The effect of positive ΔCl on CA-AKI incidence was compared with the effect of negative ΔCl for the ‘Normal Cr group’ and the ‘Slightly increased Cr group’, respectively.

### Statistical analysis

Statistical analysis was performed using SPSS for Windows, version 22.0 (SPSS Inc, Chicago, IL, USA). Continuous variables were presented as means ± standard deviations, and categorical variables were presented as numbers and percentages. Groups were compared with ANOVA for continuous variables and the χ2 test for categorical variables, respectively. Multivariate logistic regression analyses were conducted to estimate hypochloremia and hyperchloremia for the incidence of CA-AKI with relative risk (RR) and 95% confidence interval (CI). Among the significant covariates identified by univariate analysis (*P* < 0.1), several variables were selected after correlation analysis to avoid multicollinearity (variance inflation factor < 10); age, sex, admission status of patients, the presence of DM, hypertension, CAD, heart failure, serum BUN, Cr, tCO_2_. A final multiple logistic regression analysis was then conducted in a backward stepwise manner. All tests were two-sided, and a P value of < 0.05 was considered statistically significant.

## Electronic supplementary material

Supplemental Tables
