# Supplementary material for: Baseline Chloride Levels are Associated with the Incidence of Contrast-Associated Acute Kidney Injury
Source: Sci Rep. 2017 Dec 12;7:17431. doi: 10.1038/s41598-017-17763-7 (PMC5727178; doi:10.1038/s41598-017-17763-7)
Supplement: Supplementary file 1 — Supplemental Tables [file 41598_2017_17763_MOESM1_ESM.doc]

**Supplemental Table 1. Baseline characteristics of patients who underwent contrast CT with baseline serum creatinine levels of less than 1.2 mg/dL**

| **Variables** | | | **Total**  **(n=12,280, 100%)** | **Hypochloremia**  **(n=2,375, 19.3%)** | **Normochloremia**  **(n=9,697, 79.0%)** | **Hyperchloremia**  **(n=208, 1.7%)** | ***P*-value** |
| --- | --- | --- | --- | --- | --- | --- | --- |
| **Age, years** | | | 58.8±15.3 | 59.9±14.8 | 58.4±15.4 | 63.6±16.5 | <0.001 |
| **Male, n (%)** | | | 6,971 (56.8%) | 1,383 (58.2%) | 5,491 (56.6%) | 97 (46.6%) | 0.004 |
| **BMI, kg/m^2^** | | | 22.7±3.5 | 22.1±3.5 | 22.8±3.4 | 22.8±4.1 | <0.001 |
| **Inpatient, n (%)** | | | 10810 (88.0%) | 2251(94.8%) | 8355 (86.2%) | 204 (98.1%) | <0.001 |
| **Comorbid disease, n(%)** | | |  |  |  |  |  |
|  | **DM** | | 3,463 (28.2%) | 808 (34.0%) | 2,582 (26.6%) | 73 (35.1%) | <0.001 |
|  | **Hypertension** | | 5,336 (43.5%) | 1,089 (45.9%) | 4,134 (42.6%) | 113 (54.3%) | <0.001 |
|  | **Dyslipidemia** | | 2,150 (17.5%) | 413 (17.4%) | 1,692 (17.4%) | 45 (21.6%) | 0.287 |
|  | **CAD** | | 1,229 (10.0%) | 245 (10.3%) | 945 (9.7%) | 39 (18.8%) | <0.001 |
|  | **Heart failure** | | 446 (3.6%) | 88 (3.7%) | 344 (3.5%) | 14 (6.7%) | 0.051 |
| **Contrast volume, mL** | | | 120.1±20.0 | 116.7±20.8 | 121.0±19.7 | 118.0±21.9 | <0.001 |
| **Laboratory data** | | |  |  |  |  |  |
|  | | **BUN, mg/dL** | 15.4±7.8 | 16.3±8.8 | 15.0±7.3 | 22.8±13.6 | <0.001 |
|  | | **Creatinine, mg/dL** | 0.72±0.20 | 0.70±0.22 | 0.72±0.20 | 0.75±0.24 | <0.001 |
|  | | **Sodium, mEq/L** | 137.5±4.4 | 132.0±4.4 | 138.7±3.1 | 145.3±5.1 | <0.001 |
|  | | **Chloride, mEq/L** | 101.0±4.8 | 93.9±3.5 | 102.5±2.8 | 113.7±3.9 | <0.001 |
|  | | **tCO2, mmol/L** | 23.9±3.5 | 24.5±4.0 | 23.8±3.3 | 20.8±3.9 | <0.001 |
| **CA-AKI rate, n(%)** | | | 485 (3.9%) | 115 (4.8%) | 353 (3.6%) | 17 (8.2%) | <0.001 |

Data are expressed as mean (with standard deviation) or n (%)

Abbreviations; CT, computed tomography; DM, diabetes mellitus; CAD, coronary arterial disease; BUN, blood urea nitrogen; tCO_2_, total CO_2_

Hypochloremia; chloride level less than 98 mEq/L at baseline

Normochloremia; chloride level between 98 to 110 mEq/L at baseline

Hyperchloremia; chloride level over 110 mEq/L at baseline

**Supplemental Table 2. Baseline characteristics of patients who underwent contrast CT with baseline serum creatinine levels of 1.2 to 2.0 mg/dL**

| **Variables** | | | **Total**  **(n=808, 100%)** | **Hypochloremia**  **(n=150, 18.6%)** | **Normochloremia**  **(n=625, 77.4%)** | **Hyperchloremia**  **(n=33, 4.1%)** | ***P*-value** |
| --- | --- | --- | --- | --- | --- | --- | --- |
| **Age, years** | | | 68.3±13.7 | 62.3±13.3 | 63.3±13.9 | 68.4±10.9 | 0.072 |
| **Male, n (%)** | | | 664 (82.2%) | 121 (80.7%) | 520 (83.2%) | 23 (69.7%) | 0.123 |
| **BMI, kg/m^2^** | | | 23.4±3.5 | 23.2±3.9 | 23.5±3.3 | 22.7±4.0 | 0.386 |
| **Inpatient n, (%)** | | | 729 (90.2%) | 140 (93.3%) | 558 (89.3%) | 31 (93.9%) | 0.258 |
| **Comorbid disease, n(%)** | | |  |  |  |  |  |
|  | **DM** | | 337 (41.7%) | 65 (43.3%) | 259 (41.4%) | 13 (39.4%) | 0.881 |
|  | **Hypertension** | | 545 (67.5%) | 93 (62.0%) | 425 (68.0%) | 27 (81.8%) | 0.074 |
|  | **Dyslipidemia** | | 211 (26.1%) | 39 (26.0%) | 163 (26.1%) | 9 (27.3%) | 0.988 |
|  | **CAD** | | 139 (17.2%) | 21 (14.0%) | 110 (17.6%) | 8 (24.2%) | 0.317 |
|  | **Heart failure** | | 60 (7.4%) | 16 (10.7%) | 42 (6.7%) | 2 (6.1%) | 0.242 |
| **Contrast volume, mL** | | | 126.6±13.0 | 126.1±19.5 | 127.1±17.6 | 118.9±25.4 | 0.040 |
| **Laboratory data** | | |  |  |  |  |  |
|  | | **BUN, mg/dL** | 26.4±13.0 | 29.1±13.9 | 25.8±12.6 | 27.1±14.1 | 0.017 |
|  | | **Creatinine, mg/dL** | 1.41±0.19 | 1.41±0.19 | 1.40±0.19 | 1.41±0.19 | 0.810 |
|  | | **Sodium, mEq/L** | 137.2±4.9 | 131.8±5.1 | 138.1±3.6 | 144.1±5.2 | <0.001 |
|  | | **Chloride, mEq/L** | 101.6±5.4 | 93.6±3.6 | 102.9±3.2 | 113.2±2.5 | <0.001 |
|  | | **tCO2, mmol/L** | 218±4.2 | 22.8±4.6 | 21.7±3.9 | 18.8±6.0 | <0.001 |
| **CA-AKI rate, n(%)** | | | 83  (10.3%) | 22  (14.7%) | 55  (8.8%) | 6  (18.2%) | <0.001 |

Data are expressed as mean (with standard deviation) or n (%)

Abbreviations; CT, computed tomography; DM, diabetes mellitus; CAD, coronary arterial disease; BUN, blood urea nitrogen; tCO_2_, total CO_2_

Hypochloremia; chloride level less than 98 mEq/L at baseline

Normochloremia; chloride level between 98 to 110 mEq/L at baseline

Hyperchloremia; chloride level over 110 mEq/L at baseline

**Supplemental Table 3A. Incidence of CA-AKI according to the total used contrast volume (mL)**

| **Total used contrast volume (mL)** | **No CA-AKI** | **CA-AKI** | ***P*-value** |
| --- | --- | --- | --- |
| **All the enrolled patients** | 120.5±20.0 | 121.6±20.0 | 0.169 |
| **Normal Cr group** | 120.0±20.0 | 121.5±19.7 | 0.113 |
| **Slightly increased Cr group** | 127.1±18.0 | 122.3±21.3 | 0.024 |

Data are expressed as mean (with standard deviation)

Abbreviations; CA-AKI, contrast-associated acute kidney injury
